# Core non-coding RNAs of *Piscirickettsia salmonis*

**DOI:** 10.1371/journal.pone.0197206

**Published:** 2018-05-16

**Authors:** Cristopher Segovia, Raul Arias-Carrasco, Alejandro J. Yañez, Vinicius Maracaja-Coutinho, Javier Santander

**Affiliations:** 1 Marine Microbial Pathogenesis and Vaccinology Laboratory, Department of Ocean Sciences, Memorial University of Newfoundland, Logy Bay, Canada; 2 PhD Program in Integrative Genomics, Universidad Mayor, Huechuraba, Chile; 3 Laboratory of Integrative Bioinformatics, Center for Genomics and Bioinformatics, Faculty of Sciences, Universidad Mayor, Huechuraba, Chile; 4 Instituto de Bioquímica y Microbiología, Facultad de Ciencias, Universidad Austral de Chile, Valdivia, Chile; 5 Beagle Bioinformatics, Santiago, Chile; National Center for Biotechnology Information, UNITED STATES

## Abstract

*Piscirickettsia salmonis*, a fastidious Gram-negative intracellular facultative bacterium, is the causative agent o Piscirickettsiosis. *P*. *salmonis* has broad host range with a nearly worldwide distribution, causing significant mortality. The molecular regulatory mechanisms of *P*. *salmonis* pathogenesis are relatively unknown, mainly due to its difficult *in vitro* culture and genomic differences between genogroups. Bacterial non-coding RNAs (ncRNAs) are important post-transcriptional regulators of bacterial physiology and virulence that are predominantly transcribed from intergenic regions (*trans*-acting) or antisense strand of open reading frames (*cis*-acting). The repertoire of ncRNAs present in the genome of *P*. *salmonis* and its possible role in bacterial physiology and pathogenesis are unknown. Here, we predicted and analyzed the core ncRNAs of *P*. *salmonis* base on structure and correlate this prediction to RNA sequencing data. We identified a total of 69 ncRNA classes related to tRNAs, rRNA, thermoregulators, antitoxins, ribozymes, riboswitches, miRNAs and antisense-RNAs. Among these ncRNAs, 29 classes of ncRNAs are shared between all *P*. *salmonis* genomes, constituting the core ncRNAs of *P*. *salmonis*. The ncRNA core of *P*. *salmonis* could serve to develop diagnostic tools and explore the role of ncRNA in fish pathogenesis.

## Introduction

The genus *Piscirickettsia* includes two species, the recently described *P*. *litoralis* [1] and *P*. *salmonis*. *P*. *salmonis* is the etiological agent of salmonid rickettsial septicemia (SRS) or Piscirickettsiosis [2]. SRS has a high impact on the Atlantic salmon (*Salmo salar*) aquaculture in Chile, with up to ~100% of losses associated to *P*. *salmonis* infection in seawater [3]. This Gram-negative, intracellular facultative pathogen was first isolated from Coho salmon (*Oncorhynchus kisutch*) in Chile [4] and since then, it has been reported in different geographic locations (e.g. Canada, USA, Norway, UK, Greece), and isolated from different salmonid and non-salmonid species [5,6].

The *P*. *salmonis* strain LF-89 isolated in Chile is the reference strain [7,8] but many others have been isolated and characterized [9,10]. The knowledge about *P*. *salmonis* regulatory mechanisms of pathogenesis and physiology are limited due to its fastidious nature [9,11,12]. *P*. *salmonis* causes a systemic infection associated with the Dot/Icm type IV secretion system (SSTIV), which is required for cell invasion, immune evasion, and intracellular replication [13]. Also, it has been reported that *P*. *salmonis* macrophage internalization is mediated by clathrin [14]. Additionally, it has been shown that *P*. *salmonis* secretes outer membrane vesicles (OMVs) that could deliver or translocate effectors and other virulence factors into the fish cell [15]. Recently, pathogenic genomic islands have been identified in *P*. *salmonis* [16]. However, the repertoire and the potential roles of non-coding RNAs (ncRNAs) in *P*. *salmonis* gene regulation and pathogenesis have not been described.

ncRNAs are functional molecules of RNAs that are not translated into protein [17]. Genomic regions transcribed into ncRNAs, beside tRNAs and rRNAs, were not considered relevant for biological roles. The discovery of the first functional microRNA (miRNA) in *Caenorhabditis elegans* [18], claimed the scientific attention back to ncRNAs. Today, it is known that ncRNAs play important biological roles in all kingdoms of life [19, 20].

Bacterial ncRNAs are generally classified as small RNAs (sRNAs). These molecules are involved in the fine-tuning regulation of different important bacterial physiological processes. For instance, the sRNA SgrS participates in glucose uptake regulation [21], CrcZ participates in carbon catabolite repression [22], GlmY/Glm*Z* participates in feedback inhibition of amino sugar metabolism [23], and RhyB regulates the synthesis of siderophores and iron acquisition [24, 25]. sRNAs also have important roles in temperature response [26], bacterial communication [27], biofilm formation [27,28], iron metabolism [29], and virulence [30–32].

The advancement of high-throughput expression technologies over the last years boosted the prediction, characterization, and functional classification of different novel types of sRNAs [33]. This was followed by the development of several computational biology approaches, based on secondary structure predictions, sequence similarity searches, covariance analysis models, and minimum free energy models, which together allowed the identification of thousands of different RNA classes from different evolutionary branches [34, 35].

Complexity of organisms along the evolution has been associated with the expansion of genomic elements [36, 37]. Comparison between the increasing number of protein-coding genes and non-protein coding genes reveals that the expansion of ncDNA is much higher than the expansion of protein coding genes [38]. This correlates with the increasing number of sRNAs described in bacteria genomes [39].

Here we predicted the sRNAs of several *P*. *salmonis* genomes and identified the core ncRNA repertoire of *P*. *salmonis*. The ncRNAs repertoire of *P*. *salmonis* and the possible role in gene regulation and pathogenesis will contribute to understanding *P*. *salmonis* physiology and host-pathogen interaction, opening new venues for the control of this pathogen.

## Material and methods

### ncRNAs predictions in *P*. *salmonis*

The genome sequences of eleven *P*. *salmonis* strains (Table 1) were downloaded from National Center for Biotechnology Information (NCBI) [40]. The prediction was performed by comparing the secondary structures in covariance models from all RNA families available in the RNA families database (Rfam; version 12.0) [41] against the *P*. *salmonis* genome sequences (Table 1). The comparisons were performed using an in-house developed tool called StructRNAfinder [42]. This software automatically integrates different tools for ncRNAs prediction and secondary structure identification, including Infernal [43], RNAFOLD [44] and Rfam database. StructRNAfinder utilizes Infernal to generate covariance models and sequence comparisons, and RNAfold for secondary structure prediction. The functional annotation for the predicted ncRNAs is obtained from Rfam. Predicted ncRNAs overlapping the genomic coordinates of coding genes were detected using intersectBED v2.26.0 [45] and manually discarded. Also, ncRNAs predicted more than once in each *P*. *salmonis* genome were manually eliminated to reduce redundancy. Finally, ncRNAs detected in intergenic regions were considered as part of the *P*. *salmonis* ncRNA repertoire.

**Table 1:** Genomes of *P*. *salmonis* utilized in this study.

NCBI Accession	Strain	Assembly level N° Contigs	GC%
NZ_AMFF00000000	LF-89	284	40,1
NZ_ASSK00000000	LF-89	355	39,6
NZ_AZYQ00000000	AUSTRAL-005	227	39,8
NZ_JRHD00000000	T-GIM	342	39,5
NZ_JRHP00000000	EM-90	534	40
NZ_LELB00000000	FIUCHILE-89l01	285	39,8
NZ_CP011849	ATCC VR-1361	1	39,66
NZ_CP012508	PM32597B1	1	39,62
NZ_CP012413	PM15972A1	1	39,73
NZ_CP013944	PSCGR01	1	39,2
NZ_CP013975	CGR02	1	39,6

### Determination of the *P*. *salmonis* core ncRNAs

We clustered the *P*. *salmonis* genomes based on the ncRNAs repertoire of each genome. ncRNA classes were hierarchically clustered using the “complete method” and Euclidean distance through hclust function from R environment. The final heatmap representation was built using gplots R package. The ncRNAs shared by all *P*. *salmonis* genomes were considered as part of the *P*. *salmonis* ncRNA core.

### Determination of *P*. *salmonis* codon usage

The *P*. *salmonis* codon usage was determined mediated the web suite SMS (Sequence Manipulation Suite) [46]. Known functionally annotated and unique hypothetical *P*. *salmonis* proteins, based on NCBI annotation [40], were used to determine the codon usage (S1 Table).

### *P*. *salmonis* growth conditions for transcriptome analysis

The reference strain LF-89 strain was maintained and cultivated in CHSE-214 cells at 18°C [47]. From infected cells, the bacterium was streaked onto CHAB agar plates (Brain heart infusion supplemented with L-cysteine 1 gL^-1^ and 5% ovine blood) and incubated for 10 days at 18°C, until the formation of slightly convex and grey–white shiny bacterial colonies [47]. Finally, 10 single colonies were inoculated in 50 ml of Austral-SRS broth [48] and incubated for 5 days at 18°C with gentle shaking (100 rpm).

### RNA extraction and cDNA synthesis

*P*. *salmonis* grown in Austral-SRS medium was used for RNA extraction. 50 ml of bacterial culture were centrifuged (6,000 x g) during 10 min and resuspended in 1 ml of Trizol (Invitrogen, Madison, USA). The mixture was vortexed and treated with 700 μl of chloroform. The aqueous phase was extracted and mixed 1:1 with isopropanol. Total RNA was concentrated by RNeasy cleanup QIAgene kit. The total RNA extracted was treated with Turbo-DNAase I during 30 min at 37°C (Ambion). The absence of DNA was checked by PCR using the ITS primers RTS1 (5’-TGATTTTATTGTTTAGTGAGAATGA-3’) and RTS4 (5’-ATGCACTTATTCACTTGATCATA-3’) [49]. The purity was determined (ratio A260/A280) with a Nanodrop ND1000 spectrophotometer (Thermo Fisher Scientific, Copenhagen, USA), and the integrity was determined by agarose gel under denaturing conditions.

### RNA sequencing of *P*. *salmonis* LF-89

Double-stranded cDNA libraries were constructed using the TruSeq RNA Sample Preparation Kit v2 (Illumina®, San Diego, CA, USA). Two biological replicates were sequenced using the MiSeq (Illumina®) platform, at the Center for Genomics and Bioinformatics, Faculty of Sciences, Universidad Mayor, Huerchuraba, Chile. The raw sequencing reads were analyzed using CLC Genomics Workbench software, version 10.0.1 (Qiagen). The reads were trimmed using the quality score limit of 0.08 and maximum limit of 2 ambiguous nucleotides. Trimmed reads were mapped to the genome and the protein-coding genes of *P*. *salmonis* LF-89 (ATCC VR-1361; genome AMFF02000000). The expression levels were normalized and evaluated by RPKM method, as described by Mortazavi et al [50]. The raw data was made available at the NCBI SRA database [51], under the Accession number PRJNA383157.

### ncRNAs identification and expression confirmation using RNA sequencing (RNA-seq)

We used the PRJNA383157 RNA sequencing data to validate the ncRNAs predicted in the genome *P*. *salmonis* LF-89 using covariance models searches. Also, the public *P*. *salmonis* RNA-seq, PRJNA413076, PRJNA413086, PRJNA413085 and PRJNA413083 available at NCBI were utilized. The software sRNA-Detect, which was designed to identify ncRNAs from RNA-seq data [52] was utilized. sRNA-Detect search for reads that have a minimum depth coverage, with a length range corresponding to a ncRNA (< 250 bp), and a low coverage variation rate through their sequence. The input files in sequence alignment map (SAM) format were generated using Bowtie2 [53]. Predicted ncRNAs within coding regions were detected using intersectBED [45] and manually discarded as described previously. Also, we cross-referenced the genomic coordinates of the ncRNAs predicted by covariance models, against those predicted based on *P*. *salmonis* transcriptional activity through intersectBED. This step allowed us to validate the set of ncRNAs classes predicted in *P*. *salmonis* LF-89 strain using StructRNAfinder tool. Finally, the Bowtie2 alignment files were converted from sam to bam format, sorted, and indexed using SamTools [54]. These files from each RNA-seq data were visualized and compared with the Integrative Genomics Viewer (IGV) version 2.3.92 [55].

### RNA-RNA interaction

In order to identify potential target coding genes regulated by a set of selected ncRNAs predicted in *P*. *salmonis*, we used IntaRNA tool [56]. Similarly to the RNA-seq assays, we used the protein coding genes from the reference strain LF-89 (accession number: NZ_AMFF00000000) to identify the set of candidate genes potentially regulated by four selected ncRNAs (CsrC, PrrB_RsmZ, MicX and Sx4) present in the repertoire of *P*. *salmonis*. These ncRNAs were selected because they were predicted by the StructureRNAfinder and detected by the sRNA-Detect tool. Additionally these ncRNA have found in other bacterial species. We set a value of minimum energy cutoff of ΔG < -15 to be considered as potential interaction. RNA-RNA binding specificity parameters used have been previously validated in other Gram-negative bacteria such as *E*. *coli* and *Salmonella* [56–58].

## Results

### General prediction of *Piscirickettsia salmonis* ncRNAs using covariance models

**Fig 1:**
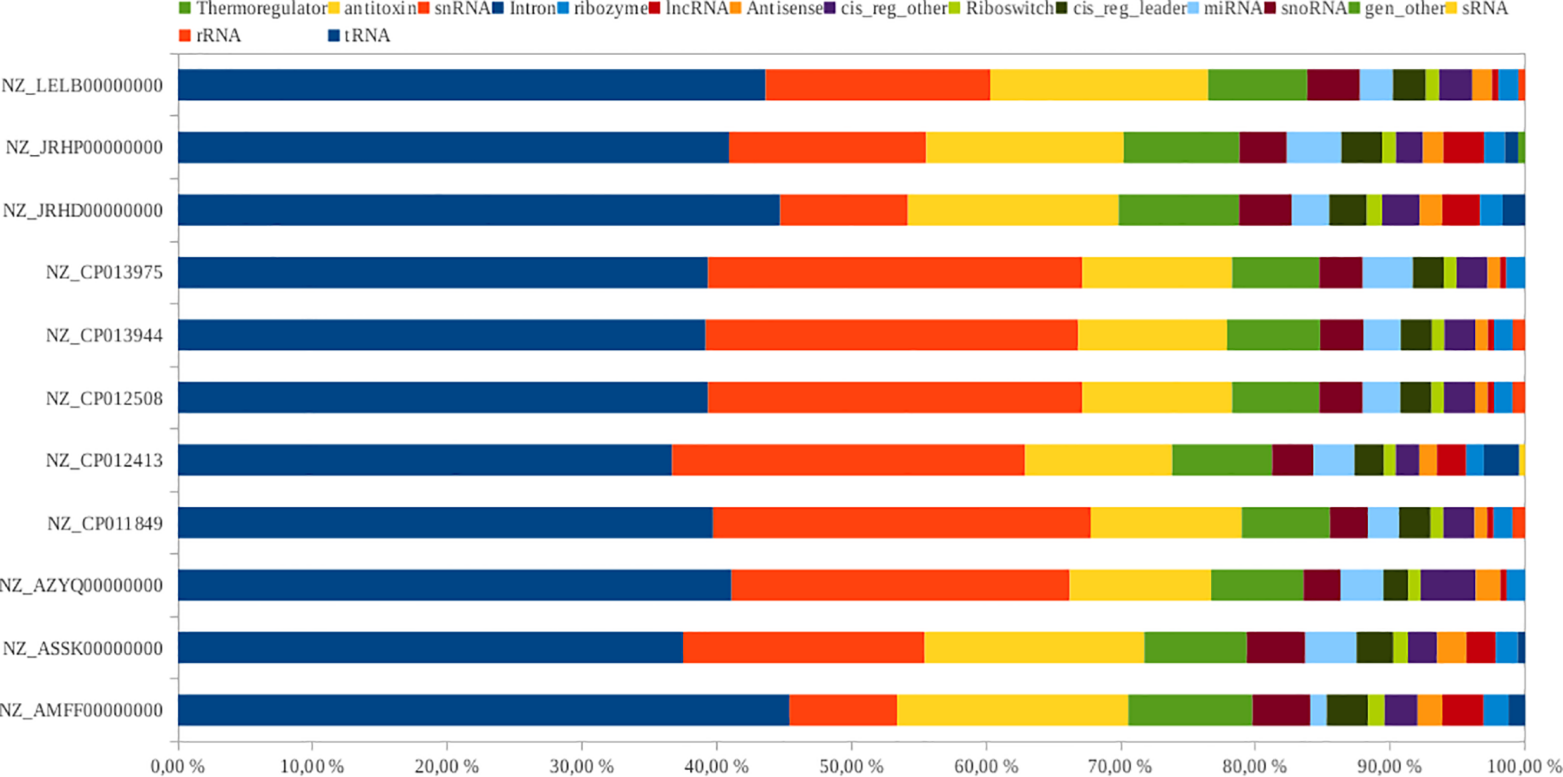
Number of ncRNA per family, the most abundant RNA families as was expected where tRNA, rRNA and sRNA. The number of rRNA in certain genomes varies attributable to the number of contigs. Also in all the analyzed genomes were predicted miRNA-like.

Sixteen RNA families were found in the eleven analyzed *P*. *salmonis* genomes. Based on covariance models, we predict 2239 ncRNAs (Fig 1). As expected, the most abundant ncRNAs families were tRNAs (40.38%) and rRNAs (21.42%). sRNAs corresponded to the 21.42%, suggesting that sRNAs play an important role in *P*. *salmonis* gene regulation. We found around 3% of miRNA-like, 1.5% of ribozymes, 1.4% of antisense RNAs and long ncRNAs, and 1% of riboswitches. The remaining ncRNAs were distributed among thirteen families, including snoRNAs, *cis* regulatory elements, catalytic intron RNAs, snRNAs, antitoxin RNAs, and thermoregulators.

### *P*. *salmonis* ncRNAs repertory

After manual depuration of the predicted ncRNA, we identified 1813 ncRNs predictions in the analyzed *P*. *salmonis* genomes (S2 Table). The most abundant classes were tRNAs, rRNAs and sRNAs. Within the *P*. *salmonis* sRNA repertory, we identify several types of sRNAs with known function. For instance, the CsrC sRNA related to carbon storage regulation in *E*. *coli* [59] and *Salmonella* Typhimurium [60], and the PrrB_RsmZ, which modulates the expression of genes related to secondary metabolism, swarming and lipase synthesis in *Pseudomonas* [61]. Also, we identified several sRNAs with unknown function, like the IsrK of *S*. Typhimurium expressed during stationary phase, and under low oxygen and Mg^+2^ conditions [62], the T44 sRNA induced during the early intracellular infection stage in *S*. Typhimurium [63], and the MicX outer membrane protein repressor of *Vibrio cholerae* [64]. Additionally, we identified sRNAs related to Gram-positive bacteria physiology, like the Sau-5971 associated to small-colony variants, and the RsaA that serves as repressor in *Staphylococcus aureus* [32,65].

We also found the ubiquitous sRNA 6S RNA that regulates the expression of sigma70-dependent genes [66] and the RimP-leader, a highly conserved motif terminator related to the maturation of the 30S ribosomal subunit [67].

Another sRNAs present in *P*. *salmonis* genomes are the Sok that is part of the toxin-antitoxin type I *hok*/*sok* system [68], the TPP riboswitch, also known as THI element [69], and the YybP-YkoY a riboswitch that directly binds Mn^2+^ [70].

Within the repertory of ncRNA we found the miRNAs-like, mir167-1, mir-821, mir-529, mir-574, mir-944, mir-458, and mir-628. miRNAs have been found in several bacterial genomes but their role during infection is not well understood [71,72].

### Determination of *P*. *salmonis* codon usage

We found that the tRNAs of *P*. *salmonis* are conserved between *P*. *salmonis* genomes. The *P*. *salmonis* codon usage showed some similarities and differences to the *E*. *coli* codon usage (Table 2). For instance, *P*. *salmonis* arginine (arg), asparagine (asn), cysteine (cys), glycine (gly), histidine (his), isoleucine (ile), lysine (lys), methionine (met), and tryptophan (trp) have similar codons usage than *E*. *coli*. In contrast, *P*. *salmonis* alanine (ala), glutamine (gln), leucine (leu), phenyl-alanine (phe), serine (ser), threonine (thr), tirosyne (try), and valine (val) have different codon usage than *E*. *coli*. In *P*. *salmonis* the most utilized condons are GCA (36%) for ala, CAA (74%) for gln, TTA (43%) for leu, TTT (84%) for phe, CCA (50%) for pro, TCA (30%) for ser, ACA (38%) for thr, TAT (83%) for tyr and GTT (42%) for val, in contrast to *E*. *coli* (Table 2). Also, the most utilized *P*. *salmonis* stop codon is TAA (60%) in contrast to TAG (60%) in *E*. *coli* (Table 2).

**Table 2:** *P. salmonis* codon usage comparison with *E. coli*.

Amino Acid	Codon	*P*. *salmonis*	*E*. *coli*	Amino Acid	Codon	*P*. *salmonis*	*E*. *coli*
Ala	GCG	**13 %**	**36 %**	Leu	TTG	**10 %**	**13 %**
Ala	GCA	**36 %**	**21 %**	Leu	TTA	**43 %**	**13 %**
Ala	GCT	**34 %**	**16 %**	Leu	CTG	**7 %**	**50 %**
Ala	GCC	**18 %**	**27 %**	Leu	CTA	**10 %**	**4 %**
Arg	AGG	**8 %**	**2 %**	Leu	CTT	**21 %**	**10 %**
Arg	AGA	**15 %**	**4 %**	Leu	CTC	**10 %**	**10 %**
Arg	CGG	**3 %**	**10 %**	Lys	AAG	**20 %**	**23 %**
Arg	CGA	**13 %**	**6 %**	Lys	AAA	**80 %**	**77 %**
Arg	CGT	**32 %**	**38 %**	Met	ATG	**100 %**	**100 %**
Arg	CGC	**29 %**	**40 %**	Phe	TTT	**84 %**	**57 %**
Asn	AAT	**74 %**	**45 %**	Phe	TTC	**16 %**	**43 %**
Asn	AAC	**26 %**	**55 %**	Pro	CCG	**10 %**	**52 %**
Asp	GAT	**73 %**	**63 %**	Pro	CCA	**50 %**	**19 %**
Asp	GAC	**27 %**	**37 %**	Pro	CCT	**30 %**	**16 %**
Cys	TGT	**49 %**	**45 %**	Pro	CCC	**10 %**	**12 %**
Cys	TGC	**41 %**	**55 %**	Ser	AGT	**25 %**	**15 %**
End	TGA	**20 %**	**7 %**	Ser	AGC	**24 %**	**28 %**
End	TAG	**20 %**	**64 %**	Ser	TCG	**2 %**	**15 %**
End	TAA	**60 %**	**29 %**	Ser	TCA	**30 %**	**12 %**
Gln	CAG	**26 %**	**65 %**	Ser	TCT	**16 %**	**15 %**
Gln	CAA	**74 %**	**35 %**	Ser	TCC	**4 %**	**15 %**
Glu	GAG	**37 %**	**31 %**	Thr	ACG	**17 %**	**27 %**
Glu	GAA	**63 %**	**69 %**	Thr	ACA	**38 %**	**13 %**
Gly	GGG	**16 %**	**15 %**	Thr	ACT	**27 %**	**17 %**
Gly	GGA	**15 %**	**11 %**	Thr	ACC	**18 %**	**44 %**
Gly	GGT	**36 %**	**34 %**	Trp	TGG	**100 %**	**100 %**
Gly	GGC	**33 %**	**40 %**	Tyr	TAT	**83 %**	**57 %**
His	CAT	**72 %**	**57 %**	Tyr	TAC	**17 %**	**43 %**
His	CAC	**29 %**	**43 %**	Val	GTG	**16 %**	**37 %**
Ile	ATA	**13 %**	**7 %**	Val	GTA	**31 %**	**15 %**
Ile	ATT	**57 %**	**51 %**	Val	GTT	**42 %**	**26 %**
Ile	ATC	**30 %**	**42 %**	Val	GTC	**12 %**	**22 %**

### *P*. *salmonis* clusterization based on ncRNAs

**Fig 2:**
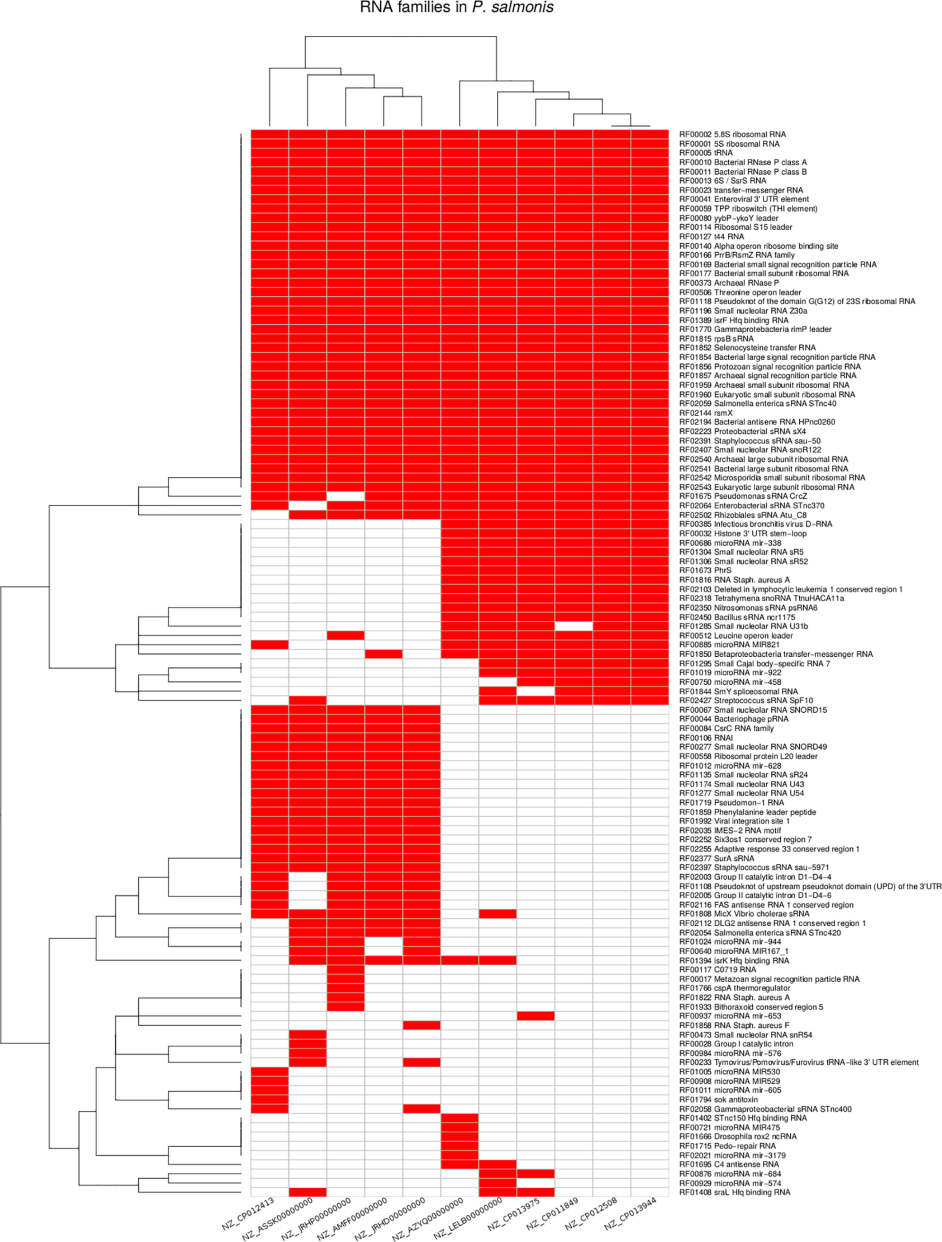
Hierarchical clustering of RNA family content in each *P*. *salmonis* strain. Presence of ncRNA classes are represented in red and the absence in white.

**Fig 3:**
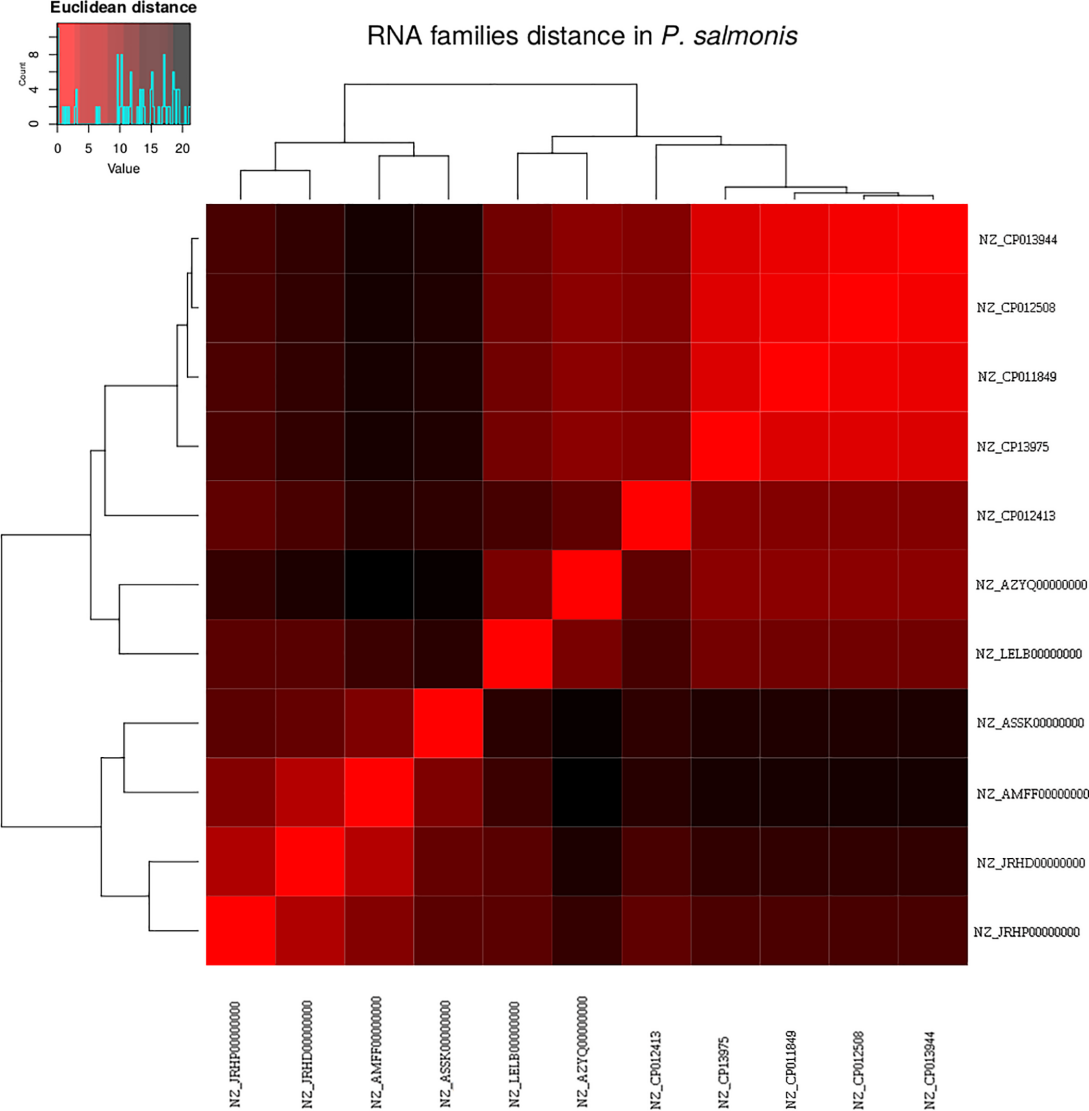
Clustering based on ncRNAs classes. Similarities between each *P*. *salmonis* strain was calculated based on Euclidean distance, using ncRNAs classes content between each *P*. *salmonis* strain are represented in each square. Low distance (in red) means a similar ncRNAs classes content and a high distance (in black) means many differences in ncRNAs classes.

The presence and absence of ncRNAs classes in the *P*. *salmonis* genomes were used to generate a heatmap representation of a hierarchical cluster through g-plots R package. The clustering was applied to both sides, one side where similar ncRNAs classes in all *P*. *salmonis* strains are clustered together, and the other side where *P*. *salmonis* strains with similar ncRNA classes are clustered together. We found that similar ncRNA clusters correlates with *P*. *salmonis* genome clusters (Fig 2). The ncRNA and the *P*. *salmonis* genomes were divided into two clusters (Fig 2). Suggesting that some ncRNAs could be strain related (Fig 3).

### ncRNAs core of *P*. *salmonis*

**Fig 4:**
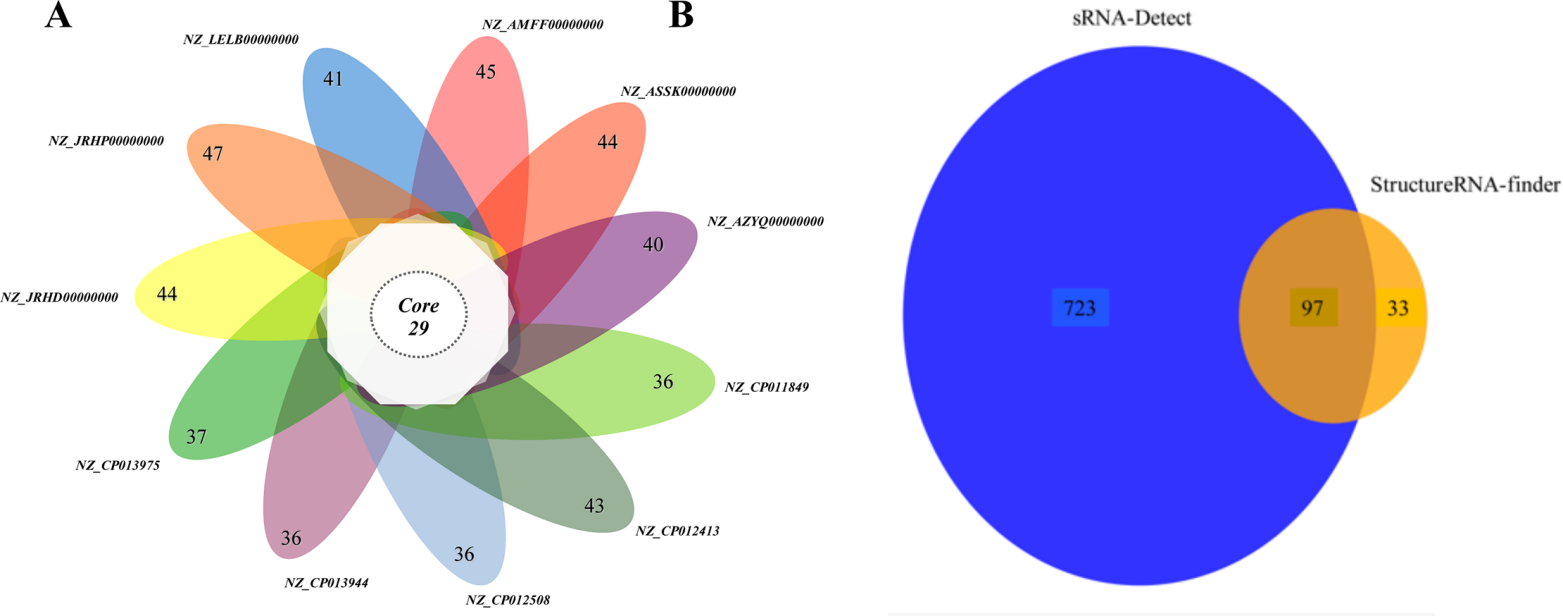
Windmill ncRNAs. **A.** Graphic representation of the ncrNAs core in *P*. *salmonis*. In middle shows the number of ncRNAs present in all genomes of *P*. *salmonis* and in the leaves are the number of ncRNAs for genome. **B.** Venn diagram between predictions by structure from StructRNAfinder and sRNA-Detected by transcriptomics analysis.

Using the ncRNA repertoire we search for the ncRNAs present in all eleven genomes. We found 29 classes of ncRNAs present in all genomes analyzed (Fig 4A), where the most abundant classes were tRNA, rRNA and sRNA with 901, 475 and 7 predictions respectively (Table 3). The sRNAs classes are reduced, in comparison with the tRNAs and rRNA, because most of these sRNAs were present only once in each genome. The T44, PrrB_RsmZ and RpsB (Rfam-RF01815) were present in a single copy per genome. Sx4 was the only one sRNA with more than one prediction per genome.

**Table 3:** ncRNA core predicted in *P*. *salmonis*.

ncRNA	Rfam ID	Characteristic/function	Presence in *P*. *salmonis* genomes
5S_rRNA	RF00001	5S ribosomal RNA	All
5_8S_rRNA	RF00002	5.8S ribosomal RNA	All
tRNA	RF00005	tRNA	All
RNaseP_bact_a	RF00010	Bacterial RNase P class A	All
RNaseP_bact_b	RF00011	Bacterial RNase P class B	All
6S	RF00013	6S / SsrS RNA	All
tmRNA	RF00023	transfer-messenger RNA	All
TPP	RF00059	TPP riboswitch (THI element)	All
yybP-ykoY	RF00080	yybP-ykoY leader	All
t44	RF00127	t44 RNA	All
PrrB_RsmZ	RF00166	PrrB_RsmZ RNA family	All
Bacteria_small_SRP	RF00169	Bacterial small signal recognition particle RNA	All
SSU_rRNA_bacteria	RF00177	Bacterial small subunit ribosomal RNA	All
RNaseP_arch	RF00373	Archaeal RNase P	All
PK-G12rRNA	RF01118	Pseudoknot of the domain G(G12) of 23S ribosomal RNA	All
rimP	RF01770	Gammaprotebacteria rimP leader	All
rpsB	RF01815	rpsB sRNA	All
tRNA-Sec	RF01852	Selenocysteine transfer RNA	All
Bacteria_large_SRP	RF01854	Bacterial large signal recognition particle RNA	All
Protozoa_SRP	RF01856	Protozoan signal recognition particle RNA	All
Archaea_SRP	RF01857	Archaeal signal recognition particle RNA	All
SSU_rRNA_archaea	RF01959	Archaeal small subunit ribosomal RNA	All
SSU_rRNA_eukarya	RF01960	Eukaryotic small subunit ribosomal RNA	All
HPnc0260	RF02194	Bacterial antisene RNA HPnc0260	All
sX4	RF02223	Proteobacterial sRNA sX4	All
LSU_rRNA_archaea	RF02540	Archaeal large subunit ribosomal RNA	All
LSU_rRNA_bacteria	RF02541	Bacterial large subunit ribosomal RNA	All
SSU_rRNA_microsporidia	RF02542	Microsporidia small subunit ribosomal RNA	All
LSU_rRNA_eukarya	RF02543	Eukaryotic large subunit ribosomal RNA	All

Also, the ribozymes RNase P class A and B [73], the riboswitches TPP and YybP-YkoY, the transcription attenuator RimP-leader, and the 6S RNA are present in all *P*. *salmonis* genomes.

### ncRNA prediction by RNA-seq

To compare our results obtained based on ncRNA structure, we analyzed the *P*. *salmonis* LF-89 transcriptome (PRJNA383157), and also the public transcriptomes of LF-89 = ATCC-VR1361 (PRJNA413076), T-GIM (PRJNA413086), S-GIM (PRJNA413085) and EM-90 (PRJNA413083) using the sRNA-Detect tool. We identified 894, 494, 619, 633, and 437 ncRNAs transcripts that correlate with the ncRNA structure prediction (S3 Table and Fig 4B), respectively. Beside tRNAs and rRNAs, the sRNAs CsrC, PrrB_RsmZ, IsrK, MicX, Sx4, and the riboswitch YybP-YkoY were identified in our RNA-seq data and in the public *P*. *salmonis* transcriptomes. For instance, the ncRNA 6S, CrcC and MicX were expressed in all *P*. *salmonis* transcriptomes analyzed (S1, S2 and S3 Figs).

### RNA-RNA interaction

**Fig 5:**
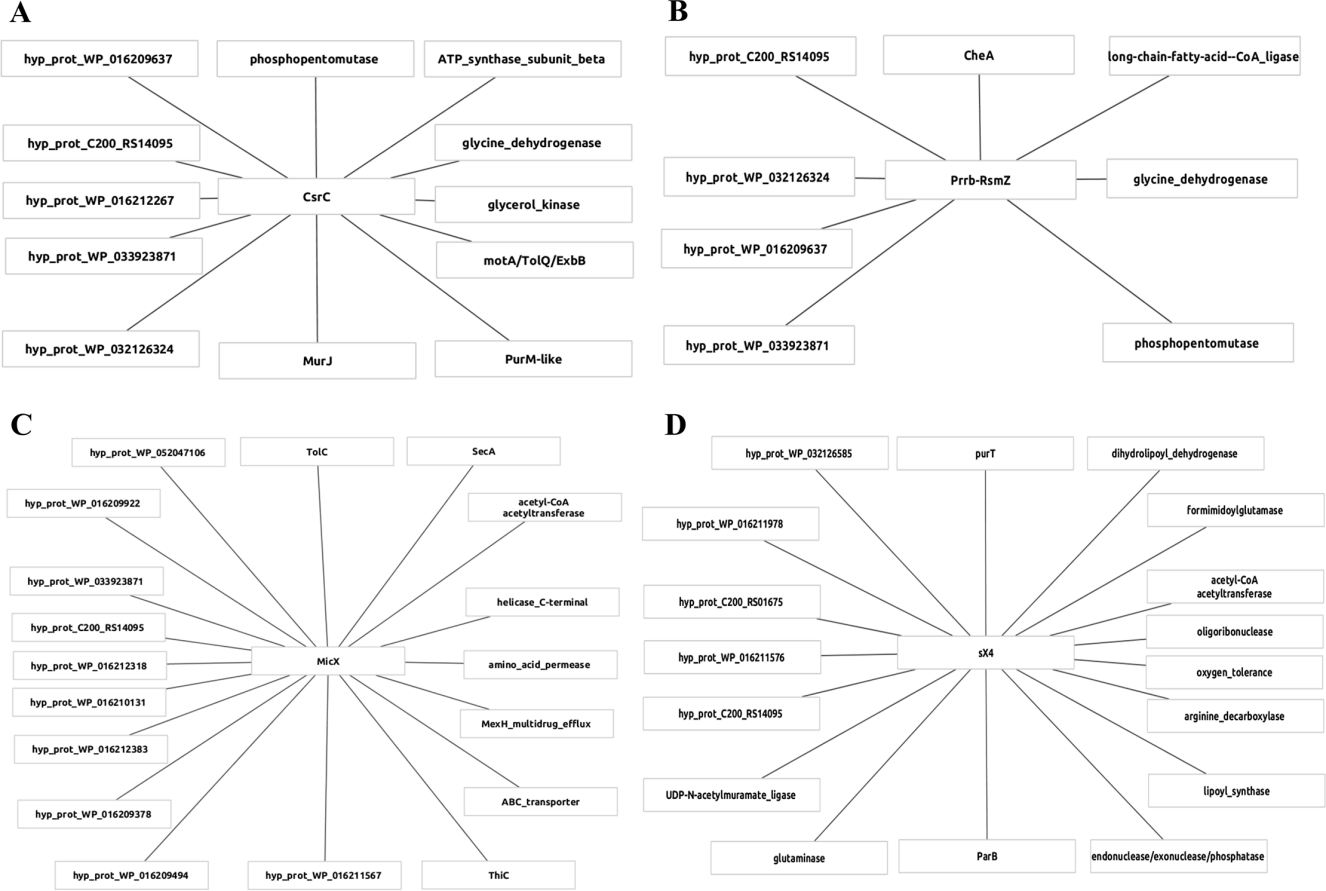
Network of RNA-RNA interactions. Potential regulatory targets with a value of minimum energy cutoff of ΔG < -15 for the ncRNAs CsrC, PrrB_Rsmz, MicX and Sx4 were plotted.

Using the IntaRNA tool, a total of 10821 possible interactions for the selected 4 ncRNAs (CsrC, PrrB_RsmZ, MicX and Sx4), with the *P*. *salmonis* coding genes were predicted without cutoff. After the cutoff (ΔG -15) was applied a total of 55 possible interactions were predicted (S4 Table, Fig 5). Forty-three percent of the 55 possible targets genes, encode for hypothetical proteins. The C200_RS14095 pseudogene is a common target for CsrC, PrrB_RsmZ, MicX and Sx4 (S4 Table). Also, we found that the gene that encode for the hypothetical protein WP_033923871 is the common target of CsrC, PrrB_RsmZ and MicX ncRNAs. CsrC and PrrB_RsmZ have 6 targets in common (S4 Table). CsrC and PrrB_RsmZ targets the genes that encode for glycine dehydrogenase (WP_016209900) and phosphopentomutase (WP_016211224; also known in *E*. *coli* as *deoB* [74]). Likely, CsrC and PrrB_RsmZ are involved in the control of metabolic pathways, related to glycine hydrogen-cyanide [75]. Another target of CsrC is *purM* gene involved in the synthesis of purine nucleotides [76]. Also, we found that CsrC targets the *murJ* gene, which is involved in the biogenesis of cell wall [77]. The proton channel proteins MotA/TolQ/ExbB that energize TonB as well flagellar rotation also are targeted by CsrC [78].

We found that PrrB_RsmZ targets the central regulator of chemotaxis CheA and biofilm [79] and the long-chain-fatty-acid—CoA ligase also known as *fadD* in *E*. *coli* [80].

Additionally our analysis showed that ncRNA MicX targets the *thiC* gene, related to methionine synthesis [81], and the gene that encode for SecA protein that is an essential component of the Type II secretion system, which has also been found in *P*. *salmonis* [82,83]. Another predicted target of MicX was the gene that encodes for the outer membrane efflux protein TolC, which is an essential functional component of the Type I secretion system [84]. Among the targets predicted for the sRNA Sx4, we found the gene that encode for the arginine decarboxylase, related to acid stress [85], the *purT* gene that encode GAR transformylase T enzyme, involved in the purine biosynthetic pathway [86], and the encoding gene of ParB protein, responsible to avoid random segregation of the plasmids prior to cell division [87].

## Discussion

Based on covariance models, we predicted 2239 ncRNAs in the eleven *P*. *salmonis* analyzed genomes. After manual depuration, 1813 ncRNAs were detected in non-coding regions and denominated as *P*. *salmonis* “ncRNA repertoire”, which consists of 69 Rfam classes (S2 Table). From this repertoire, 1383 ncRNAs (29 Rfam classes) were present in all *P*. *salmonis* genomes analyzed. These ncRNAs were considered as the *P*. *salmonis* “ncRNA core” (Fig 4A). Here we focus our discussion on the *P*. *salmonis* ncRNA core that correlates with our transcriptomic data analysis.

We found several ncRNAs that could be relevant to *P*. *salmonis* physiology, including YybP-YkoY, related to Mn^2+^ sensing response [68], and the sRNA IsrK, present in *Salmonella enterica* and *E*. *coli*, which regulates the expression of the transcriptional regulator AntQ that arrest bacterial growth [88]. Another sRNA present in the *P*. *salmonis* ncRNA core is MicX, which has been described as a regulator of genes that encoded for ABC transporters in *Vibrio cholerae* [62]. The RNA-RNA interaction analysis within the *P*. *salmonis* genome showed that MicX targets the gene that encodes for the ABC transporter substrate binding protein (WP_016210907), an orthologous of the *Vibrio sp*. ABC transporter (WP_099610902), suggesting a possible regulatory role of MicX in *P*. *salmonis* membrane transport. Additionally, we found that MicX targets the gene that encoded for the TolC protein, an essential component the Type I secretion system that plays a role in pathogenesis [89]. MicX also targets the coding gene for SecA, a Type II secretion component that is present in *P*. *salmonis* outer membrane vesicles [83, 90]. Also, RNA-seq data analysis showed that MicX is transcribed in all *P*. *salmonis* transcriptomes analyzed (S2 Fig).

The RNA-RNA interaction analysis showed that the ncRNA Sx4 could regulate the expression of the enzyme arginine decarboxylase, which plays an essential role in the tissue colonization and acid resistance during pathogenesis in enterohemorrhagic *E*. *coli* and *Shigella flexneri* [91].

The CsrC sRNA regulates the expression of the RNA-binding protein CsrA (carbon storage regulator A), a key regulatory element in bacterial carbon flux [92]. CsrA represses several processes during stationary phase, like gluconeogenesis, glycogen synthesis and catabolism [92–94]. Also, CsrA indirectly activates glycolysis and acetate metabolism during exponential phase [94,95]. CsrC sRNA sequesters CsrA protein by nine imperfect repeat sequences localized in the CsrC hairpins [59]. CsrA (WP_016209832) and CsrC ncRNA are also present in *P*. *salmonis*, reinforcing the predicted *P*. *salmonis* ncRNAs (S2 Table) and transcriptomics analyses (S3 Table).

**Fig 6:**
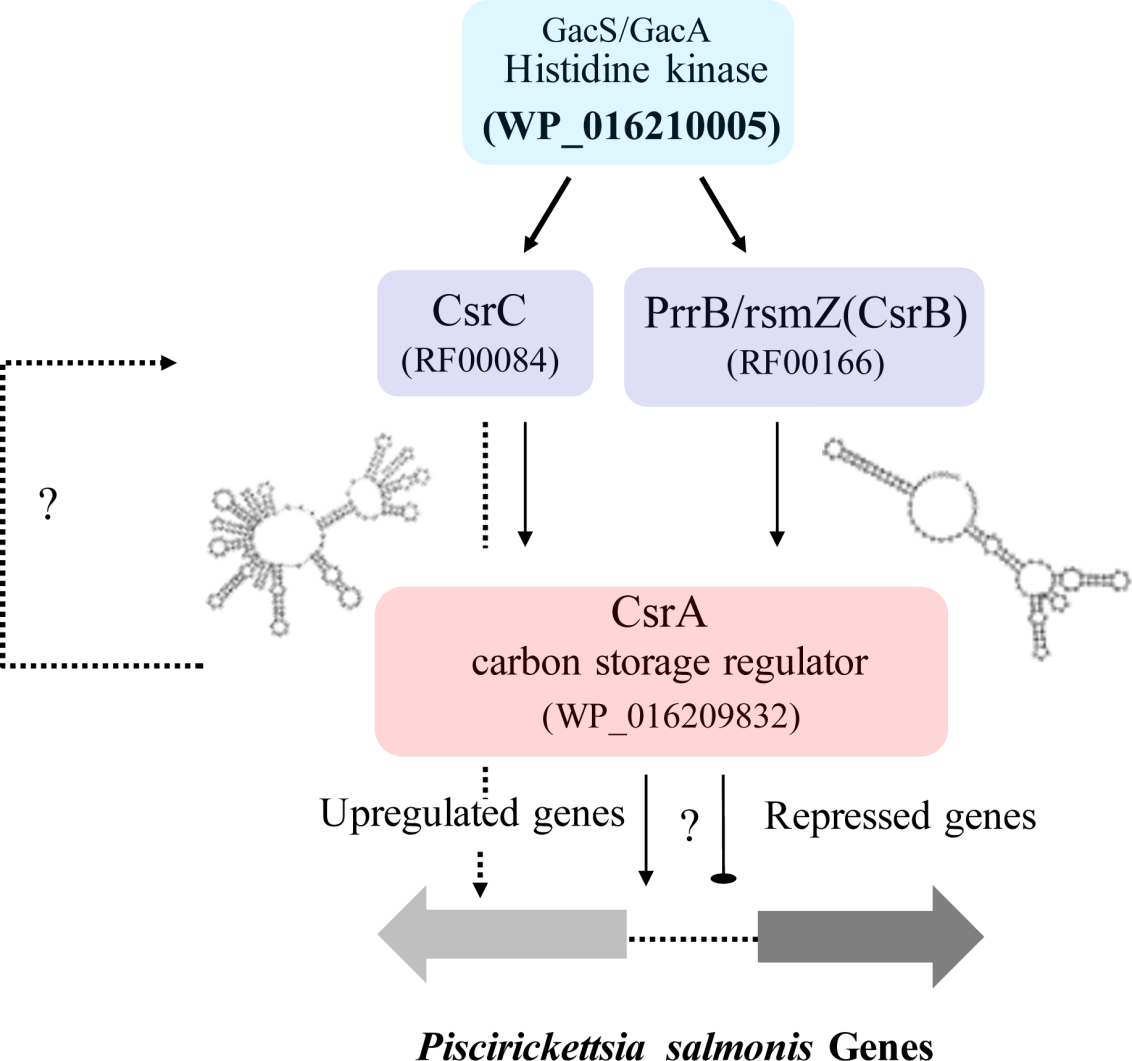
Predicted *P*. *salmonis* GacS/GacA-CsrA/CsrB/CsrC regulatory system.

Additionally, CsrA has a high identity to RsmA, a post-transcriptional regulatory protein present in *Pseudomonas aeruginosa*, *P*. *fluorescens* CHA0, and *Erwinia carotovora* [96, 97]. RsmA have global regulatory effects in *P*. *aeruginosa*, modulating *pvdS* (Iron-regulated sigma factor), *vfr* (transcriptional regulator) and *pilM* (type 4 fimbrial biogenesis protein) transcription levels [98,99]. RmsA is regulated by the two-component system GacS/GacA, also present in *P*. *salmonis*. It has shown that the GacS/GacA regulates RsmA/RsmB in *E*. *carotovora*, and CsrA/CsrB/CsrC in *E*. *coli* and *S*. *enterica* [59, 96,100, 101]. CsrC is part of the CsrB-CsrC sRNAs regulatory system of *E*. *coli* [59, 102]. CsrB has similar functions to CsrC but it differs in the number of imperfect repeat sequences that serve as a binding site to CsrA [59]. Both CsrA and CsrB indirectly activate CsrA via the response regulator UvrY9 [59]. We did not found a CsrB orthologue in *P*. *salmonis*, however, we identified the PrrB_RsmZ sRNA, a *P*. *aurigenosa* orthologue that has similar structure and function than CsrB [59,61]. The CsrB/CsrC system is also involved in pathogenesis, for instance, *Salmonella enterica* mutants of CsrC have a reduced cell invasion ability and expression of SPI1 (*Salmonella* pathogenicity island 1) related genes, and the double mutant of CsrB/CsrC is deficient for cell invasion [103]. These results suggest that the *P*. *salmonis* GacS/GacA-CsrA/CsrB/CsrC regulatory system (Fig 6) could have an important role in *P*. *salmonis* physiology and pathogenesis. However, despite the presence of this system and its possible target genes in *P*. *salmonis* genome, CsrC and PrrB_RsmZ did not show a strong interaction with the *csrA P*. *salmonis*, having a value under the defined ΔG< -15 cutoff for a strong interaction. Nevertheless, we found a strong interaction between CsrC and the proton channel MotA/TolQ/ExbB encoding gene. MotA/TolQ/ExbB energizes TonB and flagellar rotation motor, both relevant for pathogenesis, especially TonB that is required for iron acquisition [104]. Furthermore, we found that CsrC is present in all transcriptomes analyzed and shows a high transcriptional activity suggesting an important role in *P*. *salmonis* (S3 Fig).

It has been described that most of the *P*. *salmonis* isolates harbour 3–4 cryptic plasmids [105]. These results correlate with the strong predicted interaction between Sx4 sRNA and the ParB encoding gene. Also, Sx4 is the only *P*. *salmonis* core sRNA present in more than copy. Perhaps, Sx4 sRNA play a role during cell division, regulating the expression of ParB, responsible to avoid random segregation of the plasmids prior to cell division.

This is the first description of the ncRNA present in *P*. *salmonis* genome. The different ncRNA families present in different *P*. *salmonis* isolates could be utilized to determine the geographic origin, the virulence of a specific isolate or as targets for novel antibacterial treatments. The abundant number of ncRNAs predicted in the genome of *P*. *salmonis* suggest that these genetic elements play an important role in physiology and pathogenesis. However, all those predicted ncRNA targets and regulatory circuits in *P*. *salmonis* need experimental validation. Unfortunately, the genetic tools for *P*. *salmonis* are not developed yet to generate the mutant to test the effects on physiology and pathogenicity. However, despite the lack of specific genetics tools for *P*. *salmonis*, it has been reported functional validation of predicted genes through heterologous expression [106]. These assays could be a good approach to test our predictions especially to test the function by conserved secondary structure in *P*. *salmonis* ncRNAs.

## Supporting information

S1 Table Protein used to determine codon usage. (XLS)

S2 Table Repertoire of ncRNAs in *Piscirickettsia salmonis*. (XLS)

S3 Table Prediction of ncRNA by RNAseq. (XLS)

S4 Table RNA-RNA interaction against *Piscirickettsia salmonis*. (XLSX)

S1 Fig Visualization of ncRNA 6S transcription in *P*. *salmonis* transcriptomes. (TIF)

S2 Fig Visualization of ncRNA MicX transcription in *P*. *salmonis* transcriptomes. (TIF)

S3 Fig Visualization of ncRNA CsrC transcription in *P*. *salmonis* transcriptomes. (TIF)
