# Supplementary figures and images for: Core non-coding RNAs of Piscirickettsia salmonis
Source: PLoS One. 2018 May 16;13(5):e0197206. doi: 10.1371/journal.pone.0197206 (PMC5955585; doi:10.1371/journal.pone.0197206)

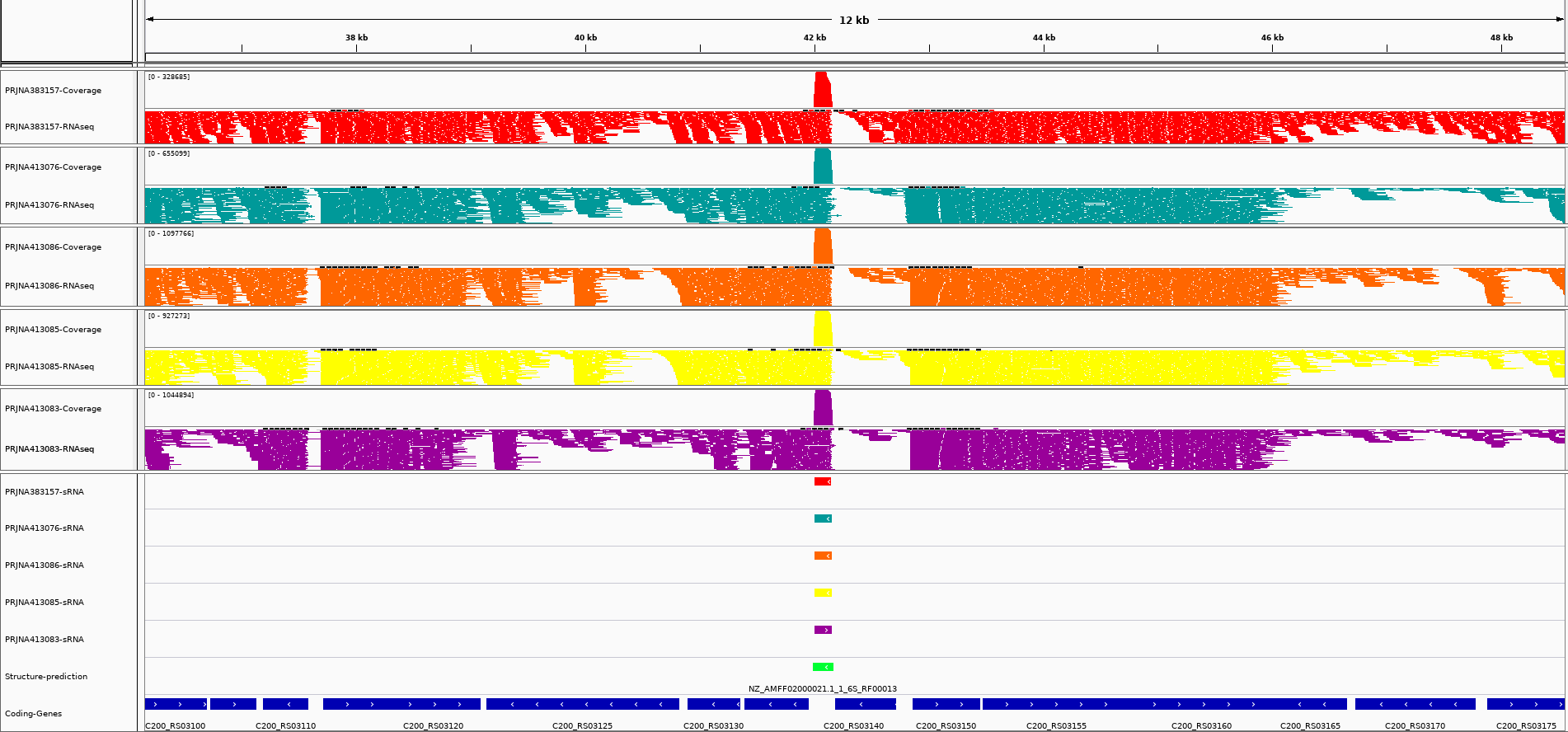

Supplement: S1 Fig — (TIF) [file pone.0197206.s005.tif]

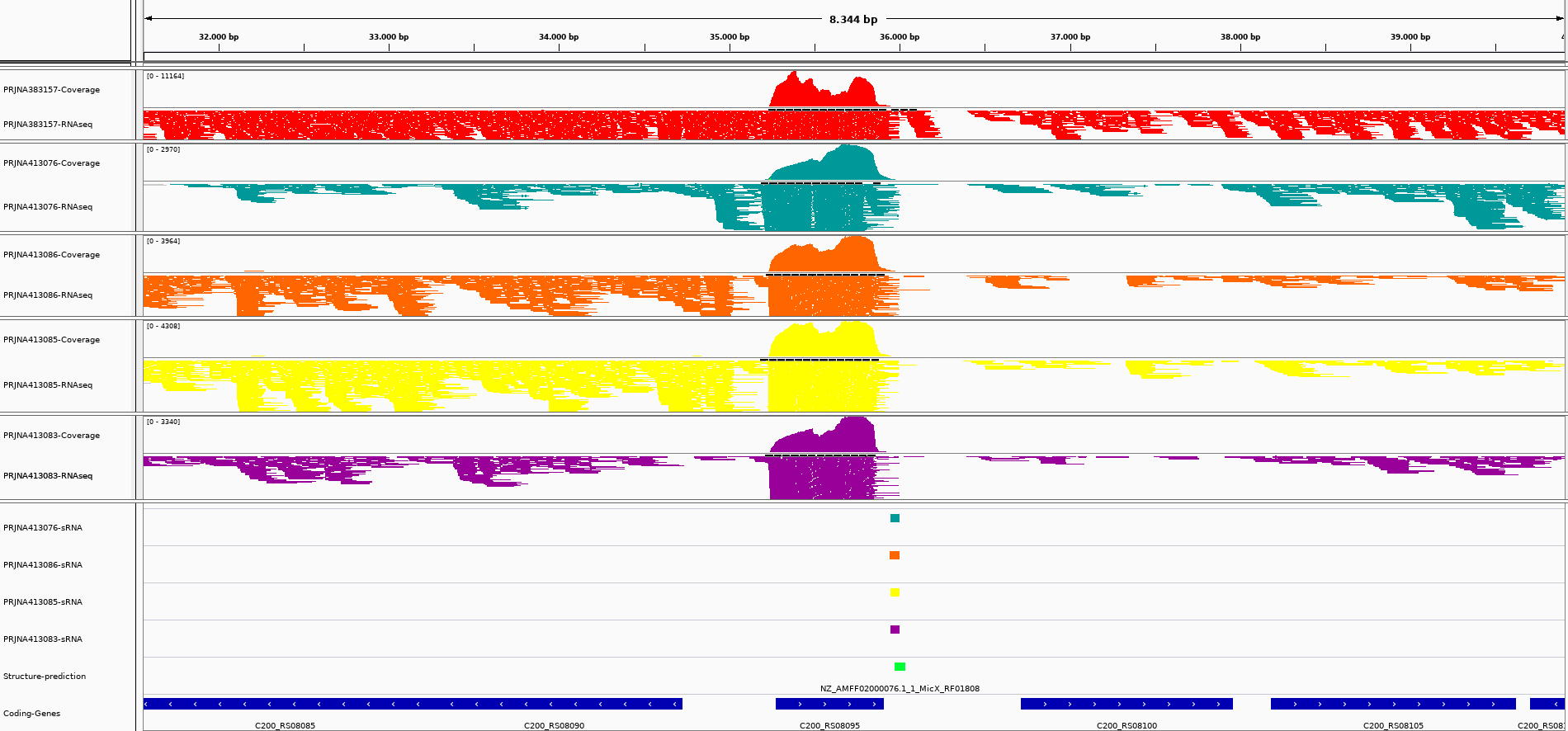

Supplement: S2 Fig — (TIF) [file pone.0197206.s006.tif]

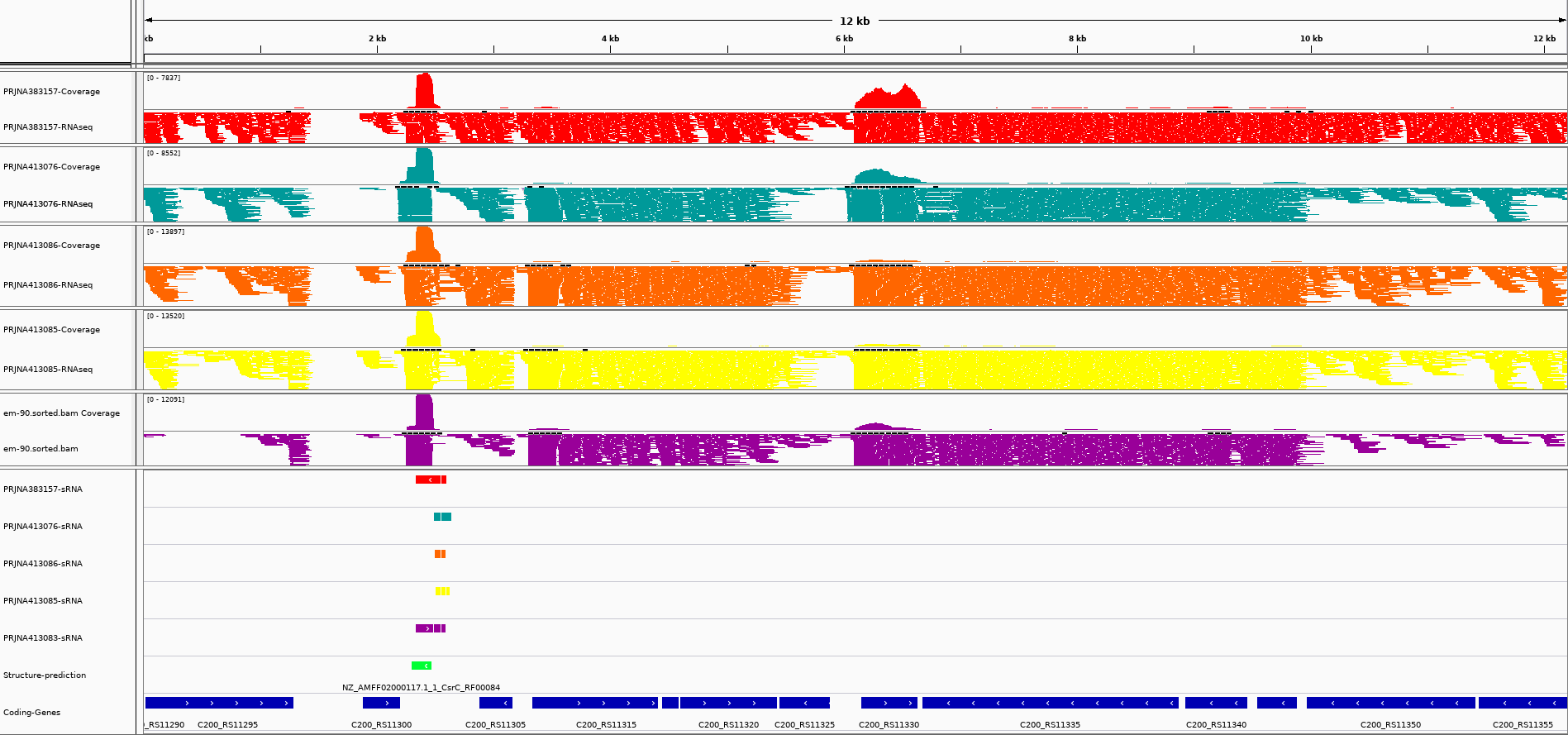

Supplement: S3 Fig — (TIF) [file pone.0197206.s007.tif]
